# Investigation of the Antimicrobial Resistance of Important Pathogens Isolated from Poultry from 2015 to 2023 in the United States

**DOI:** 10.3390/pathogens13110919

**Published:** 2024-10-22

**Authors:** Asher T. Wang, Liya Tang, Andrew Gao, Ethan Zhang, Grace Huang, Justin Shen, Qian Jia, Zuyi Huang

**Affiliations:** 1 Department of Chemical and Biological Engineering, Villanova University, Villanova, PA 19341, USA; ashertwang@gmail.com (A.T.W.); liyatang8818@gmail.com (L.T.); andrewdinggao@gmail.com (A.G.); ez0906123@gmail.com (E.Z.); gracehuang1768@gmail.com (G.H.); justinxshen@gmail.com (J.S.); 2 Department of Health, Nutrition and Exercise Sciences, Immaculata University, Immaculata, PA 19345, USA

**Keywords:** antimicrobial resistance, poultry, chicken, turkey, foodborne pathogens, antimicrobial resistance genes, principal component analysis, hierarchical clustering, time profiles, public health

## Abstract

Foodborne pathogens cause around 47.8 million illnesses in the U.S. annually, with antimicrobial misuse in food production, particularly in poultry processing, contributing significantly to this public health challenge. Misuse of antimicrobials can contribute to antimicrobial resistance (AMR) and make the treatment of pathogens increasingly difficult. This emphasizes the need to investigate antimicrobial resistance in U.S. poultry. This study analyzes data from the NCBI Pathogen Isolates Browser (2015–2023) to explore the relationships between antimicrobial-resistant pathogens, AMR genes, and antimicrobials detected with resistance in pathogens isolated from chicken and turkey. Using principal component analysis and hierarchical clustering, we mapped and profiled regional and temporal patterns of antimicrobial resistance. *Salmonella enterica* was the most prevalent antimicrobial-resistant pathogen across both chicken and turkey, with notable outbreaks, particularly in the Northeast. Antimicrobial-resistant *Campylobacter jejuni* was more prevalent in chicken, particularly in California and Georgia, while *Escherichia coli* and *Shigella* were more prominent in turkey, with concentrated antimicrobial resistance in Texas for pathogen samples isolated from chicken. Resistance to tetracycline and streptomycin was widespread, with distinct regional clusters: antimicrobial resistance was concentrated in states like Minnesota for pathogens isolated from chicken, while AMR found in pathogens isolated from turkey was more evenly distributed across the Midwest. Key AMR genes, such as *tet(A)*, *mdsA*, and *mdsB*, also followed similar patterns, peaking in 2019 and significantly declining by 2022. The observed decline in AMR cases may be linked to improved biosecurity measures and disruptions in detection due to the COVID-19 pandemic. This comprehensive study of antimicrobial resistance in U.S. poultry provides valuable insights into resistance trends, which provide useful information to inform targeted interventions and policies to mitigate AMR threats in the poultry production industry. For consumers, these findings emphasize the importance of proper food handling and cooking practices to reduce the risk of exposure to resistant pathogens. Regulatory authorities should focus on enforcing stricter antimicrobial usage policies and enhancing surveillance systems to sustain the reduction in AMR cases.

## 1. Introduction

Foodborne pathogens are a significant public health concern, with the Centers for Disease Control and Prevention (CDC) estimating an annual toll of 47.8 million illnesses, 127,839 hospitalizations, and 3037 deaths in the U.S. due to foodborne diseases [1]. This crisis not only impacts public health but also imposes substantial economic burdens, with an annual healthcare cost of USD 4.6 billion in the U.S. estimated by the CDC [2]. If current antimicrobial usage practices persist, the global economic toll of antimicrobial resistance (AMR) infections could reach a staggering USD 1.7 trillion by 2050 [3]. Antimicrobial resistance complicates the treatment of infectious diseases. AMR arises when bacteria, viruses, fungi, and parasites evolve to resist the effects of medications that were once effective against them. In the case of poultry, AMR is mainly carried by pathogen like *Salmonella*, *Campylobacter*, and *Escherichia coli*, which cause illnesses ranging from mild gastroenteritis to severe conditions such as meningitis. From 1998 to 2012, the U.S. Foodborne Disease Outbreak Surveillance System reported 1114 outbreaks, of which 279 (25%) were linked to poultry, accounting for the highest number of outbreaks, illnesses, and hospitalizations, and the second-highest number of deaths [4]. Additionally, the recent emergence of multidrug-resistant *Salmonella Infantis* (ESI) clones in poultry, driven by the pESI plasmid, poses a growing threat to human health, as these strains have evolved mechanisms to overcome common antimicrobials, with varying specific resistances across different strains [5,6]. A study analyzing poultry-associated outbreaks from 1998 to 2012 found that *Salmonella enterica* was responsible for 43% of confirmed outbreaks, with poultry being a significant source. 

A major factor exacerbating the issue of AMR is the widespread use of antimicrobials in agriculture, particularly within the livestock and poultry industries. This practice has significantly contributed to the emergence of antimicrobial-resistant pathogens, with the inappropriate use of antimicrobials in poultry farming accelerating the development of resistant strains [7,8]. Unregulated practices, such as those in backyard poultry operations, have further amplified public health concerns, as these environments often encounter wildlife, which can transmit persistent pathogens through eggs [9]. Unauthorized antimicrobial use is another critical factor driving AMR [10,11]. The lack of standardized treatment protocols and insufficient training among agricultural workers exacerbate this problem, as antimicrobials are frequently used not only to treat sick animals but also for growth promotion and disease prevention, thereby accelerating the emergence of resistant strains. Inappropriate antimicrobial selection, dosing, and treatment durations contribute to this issue, with constant use promoting the development of AMR [12,13]. Furthermore, the natural evolution of antimicrobial genes among bacteria continues to pose challenges, as AMR develops over time [14]. The presence of antimicrobials exerts constant pressure on bacterial populations, enabling those with resistant strains to survive, reproduce, and pass down resistant genes. The production of antimicrobial resistance genes (ARGs) within a population creates strong selective pressure for other bacteria to develop AMR while antimicrobials remain at sublethal levels [15]. ARGs are notably more prevalent in animal waste compared to other sources like hospitals, soil, and groundwater [15]. These resistance genes employ mechanisms to negate the effects of antimicrobials and have become increasingly common in recent studies [16]. The entry of these bacteria into human systems—whether through the consumption of contaminated products, direct contact with animals, or other means—poses a serious threat to public health.

Since AMR is recognized as a One Health issue, affecting humans, animals, and the environment, it requires integrated control policies. The key components of these policies include the surveillance of resistant bacteria and the regulation of antimicrobial use [17]. Veterinarians have highlighted the importance of adhering to treatment guidelines, along with implementing biosecurity measures and maintaining hygiene, to mitigate the effects of AMR [18]. To address the AMR threat, the U.S. National Action Plan for Combating Antimicrobial-Resistant Bacteria (CARB) was launched in March 2015. Subsequently, organizations like the American Association of Veterinary Laboratory Diagnosticians (AAVLD) established specialized resistance working groups focused on enhancing surveillance efforts and developing standardized protocols for AMR testing in diagnostic laboratories [19]. These initiatives aimed to improve the detection and monitoring of resistant bacteria in animal populations, aligning with the One Health approach to combating antimicrobial resistance. The U.S. Centers for Disease Control and Prevention (CDC), the U.S. Department of Agriculture (USDA), and the Food and Drug Administration (FDA) have jointly monitored the development of AMR in food through the National Antimicrobial Resistance Monitoring System (NARMS), an integrated surveillance system that tracks foodborne pathogens in retail meats, food animals, and human patients [19].

While existing AMR surveillance programs are valuable, some data remain inaccessible due to industry restrictions [20]. This is particularly true for data collection from U.S. poultry, which has been insufficient. The U.S. Poultry and Egg Association has released reports on “Antimicrobial Stewardship” aimed at promoting responsible antimicrobial use [21]. However, there are currently few studies that utilize databases to examine specific resistance genes across large geographic areas [16,22,23,24,25,26]. Comprehensive and detailed surveillance of AMR patterns is therefore essential for understanding AMR trends in U.S. poultry. One valuable resource for such surveillance is the National Center for Biotechnology Information (NCBI) Pathogen Isolates Browser, which analyzes bacterial pathogens from patients, food, and environmental sources. This database holds significant potential for monitoring AMR trends, as demonstrated by several existing studies [25,26].

In the European Union, the implementation of regulations within member states has resulted in a more than 50% reduction in antimicrobial consumption [27]. The lack of similar policies in the U.S. may be contributing to the overuse of antimicrobials. According to the FDA, the sale and distribution of antimicrobials in livestock decreased by 33% from 2016 to 2017 when antimicrobial sales were halted [28]. However, since 2017, antimicrobial use has been on the rise again. This study focuses on analyzing the AMR trends in 6966 isolates from U.S. poultry, primarily chicken and turkey, in the last decade (mainly from 2015 to 2023, as the data for 2024 are not complete), as recorded in the NCBI Pathogen Isolates Browser. Multivariate statistical analysis methods are employed to extract data on the genes, pathogens, and antimicrobials with detected resistance in U.S. poultry across different states over time. The findings from this research offer valuable insights for comprehensive policy reform in the U.S. to address the AMR crisis effectively. Additionally, the knowledge gained will help control public health risks and manage healthcare costs by guiding the appropriate use of antimicrobials.

## 2. Materials and Methods

**Figure 1:**
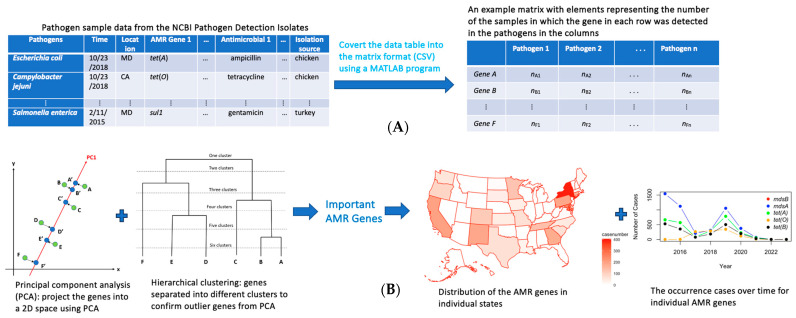
An overview of the materials and methods using AMR genes as an example: (**A**) AMR data were extracted from the NCBI Pathogen Isolates Browser and arranged into a matrix; (**B**) principal component analysis (PCA) was conducted to arrange the high-dimensional AMR genes (e.g., Gene *A* to Gene *F*) into two dimensional space so that hierarchical clustering could be used to further investigate the relations among the AMR genes and especially to identify the outlier genes presented in PCA plots—which were generally important AMR genes. The distribution of these genes in individual states was plotted for spatial patten recognition. The cases over time, i.e., the time profile, were plotted for those genes important for studying the occurrence trends. These analysis steps were conducted for data from chicken and turkey separately, for comparison.

Figure 1 provides an overview of the materials and methods used in this work, while more detail will be given in the following subsection. AMR data for U.S. poultry, specifically chicken and turkey, were obtained from the NCBI Pathogen Isolates Browser for the period from 2015 to 2023. The focus of this study was on pathogens that demonstrated resistance to at least one antimicrobial. A MATLAB program was developed to extract and organize key information from the downloaded data, including the sampling time, location, source (chicken or turkey), specific pathogens, AMR genes, and the antimicrobials to which resistance was detected. This extracted information was systematically arranged into a matrix format, where each row represented an individual isolate and each column captured specific details such as the sampling time, pathogen type, resistance genes, and antimicrobials.

The organized matrix was then analyzed using multivariate statistical methods, including principal component analysis (PCA) and hierarchical clustering. These methods were instrumental in identifying the most significant pathogens, AMR genes, and antimicrobials with detected resistance in poultry samples. PCA facilitated the reduction of data complexity by highlighting the primary components contributing to the variation in resistance profiles, while clustering analysis helped to group similar resistance patterns, revealing key relationships within the data.

To further interpret the findings, this study employed visual representations such as bar plots, geographical maps, and temporal profiles. These visual tools effectively illustrated the spatial distribution and temporal trends of the identified important pathogens, AMR genes, and resistance profile of an antimicrobial. By integrating these chemometric techniques with comprehensive data visualization, this study provided a detailed analysis of AMR in U.S. poultry to provide valuable insights into resistance patterns and potential regional hotspots.

### 2.1. Materials

The data utilized in this study were sourced from the NCBI Pathogen Isolates Browser, which is one of the most comprehensive AMR databases in the world. To ensure the relevance and specificity, several filters were applied, with a focus on the organism groups (pathogens, antimicrobials with detected resistance, and AMR genes), isolation sources (chicken and turkey), collection time, and geographic locations. These filters enabled a more targeted analysis of pathogens with detected antimicrobial resistance in poultry, specifically limiting the scope to chicken and turkey, which are critical in both agricultural production and food safety, especially in the United States. By excluding other poultry types, such as ducks, quail or geese, the analysis remained concentrated on the most significant poultry sources. In particular, chicken and turkey are the most consumed poultry sources in the U.S., meaning that focusing on antimicrobial resistance in these sources particularly will be most beneficial in examining AMR causes and issues for the next step actions. It turns out that samples sourced from the NCBI Pathogen Isolates Browser mainly contain *S. enterica*, *E. Coli* and *Shigella*, and *C. jejuni*. The antimicrobial agents detected with resistance in these foodborne pathogens in the extracted data include amoxicillin-clavulanic acid, ampicillin, azithromycin, cefoxitin, ceftiofur, ceftriaxone, chloramphenicol, ciprofloxacin, ciprofloxacin, clindamycin, erythromycin, florfenicol, gentamicin, kanamycin, meropenem, nalidixic acid, streptomycin, sulfamethoxazole, sulfisoxazole, telithromycin, tetracycline, and trimethoprim-sulfamethoxazole. The timeframe of 2015–2023 was selected as it includes significant policy actions, including the launch of the U.S. National Action Plan for Combating Antibiotic-Resistant Bacteria (CARB) in 2015 [29], which greatly enhanced antimicrobial resistance (AMR) monitoring efforts in sectors like poultry production. By focusing on the 2015–2023 period, this study aims to evaluate the effectiveness and long-term impacts of these AMR policies. There are a total of 6966 pathogen isolates with detected resistance to at least one antimicrobial agent. Although the NCBI database provides global data, this study focused exclusively on U.S. data to maintain a clear emphasis on the U.S. poultry processing industry. After extraction, the data were organized into comprehensive tables that included details about the organisms present, collection dates, sources (chicken or turkey), states where the samples were collected, and specific pathogens, antimicrobials with detected resistance, and AMR genes involved. A binary system was used to track the presence, absence, or resistance of each variable, where a value of “1” indicated presence or resistance, and a value of “0” signified absence. Figure 1A illustrates an example of the extracted matrix used in this study. This structured format facilitated the application of statistical data analysis to uncover trends, correlations, and patterns within the dataset.

### 2.2. Methods

The organized data were subjected to several analytical techniques to identify patterns and trends in antimicrobial resistance. To further create the matrices for the analysis, the subjects (e.g., AMR genes) were placed in rows while measures used to categorize the subjects (i.e., pathogens carrying the AMR gene) were placed in columns. The unique organisms present in the dataset were indexed. The matrix was organized so that the subjects in the rows (e.g., AMR genes) were characterized by the features represented in the columns (e.g., pathogens). Matrices were created for chicken and turkey, separately, for comparison. PCA is a statistical method used to reduce data dimensionality. The technique transforms high-dimensional data into a lower-dimensional space while retaining most of the information [29]. It seeks linear combinations of original variables, known as principal components, that capture maximal variance with minimal information loss [30]. This reduction in dimensionality is crucial for simplifying complex datasets for better visualization and subsequent computations [31]. On the other hand, clustering involves grouping data points into clusters based on their similarities. Clustering is essential for pattern recognition, anomaly detection, and data compression. It helps by identifying natural groupings within the dataset for better decision-making and understanding of complex data relationships [32].

The need for PCA and hierarchical clustering arises from the increasing complexity and dimensionality of our data. Applying PCA can reduce the dimensionality of data while preserving important information. This facilitates easier interpretation and analysis of datasets, which is particularly useful in identifying key outliers, such as genes, that play a significant role in antimicrobial resistance [31]. Although PCA can represent items, such as the 168 genes in this dataset, in a two-dimensional space, some genes may be lumped together, making them difficult to distinguish. Hierarchical clustering, on the other hand, allows the identification of patterns and structures that may not be apparent initially, aiding in data exploration and knowledge discovery [32]. The results were thereby plotted to visualize the distribution of AMR genes with detected resistance in the first two principal component spaces (the PC1–PC2 space). Based on this, dendrograms were then created by hierarchical clustering, and the genes were separated into different clusters to identify groups with similar occurrence patterns. The number of clusters required to identify genes of high importance was determined by the elbow method [33]. The outlier genes shown in the PC1–PC2 space were generally grouped together in the same clusters. This facilitates identification of genes important to antimicrobial resistance.

Although the previous section and Figure 1B use genes to illustrate our statistical data analysis methods, these approaches were also applied to identify pathogens and antimicrobials significantly involved in antimicrobial resistance in U.S. poultry. To further validate the significance of the identified AMR genes, the occurrence frequency of each gene was calculated separately for chicken and turkey. The genes with the highest occurrences were visualized using bar plots and compared with the top genes identified through PCA and hierarchical clustering. This method was also applied to validate the importance of the identified pathogens and antimicrobials associated with the detected resistance.

To analyze the geographical distribution of antimicrobial resistance, the state abbreviations and corresponding indices were mapped. The counts of the pathogens and top genes/antimicrobials were calculated for each state and visualized on the maps. Time profile graphs were created to illustrate the development and evolution of the top AMR genes/pathogens/antimicrobials over time. This approach involved plotting the number of cases for specific genes in poultry over a range of years. This provided insights into the trends and potential shifts in gene prevalence. By observing the trends and AMR development patterns through these profiles, periods of increased or decreased AMR prevalence can be identified. The correlation of the results with external factors such as changes in agricultural practices, introduction of new antimicrobials, and implementation of AMR management strategies may aid in combatting AMR outbreaks in the future.

## 3. Results

### 3.1. Pathogens Involved in Antimicrobial Resistance Detected in U.S. Poultry

**Figure 2:**
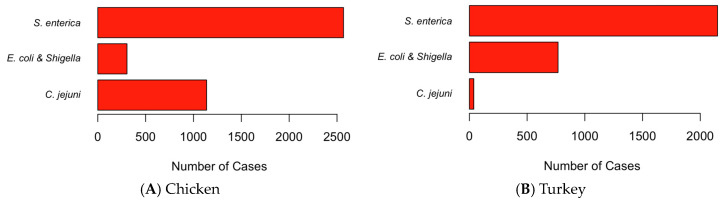
The number of cases of pathogens in chicken (**A**) and in turkey (**B**) in the U.S. by pathogen. Represents all the data in sample set.

During the 2015–2023 sample time frame, *S. enterica* was most commonly found in both chicken and turkey, present in 64.04% and 72.73% of cases reported, respectively (Figure 2). The other pathogens analyzed in this study, *C. jejuni*, and *E. coli* and *Shigella*, were also present in chicken and turkey samples, albeit to varying degrees. In chicken, *C. jejuni* was more common, detected in 28.35% of the cases. However, in turkey samples, *C. jejuni* was found in only 1.29% of cases reported. *E. coli* and *Shigella* were found much more commonly in turkey samples, in 25.98% of cases. In chicken, *E. coli* and *Shigella* were only responsible for 7.61% of cases.

**Figure 3:**
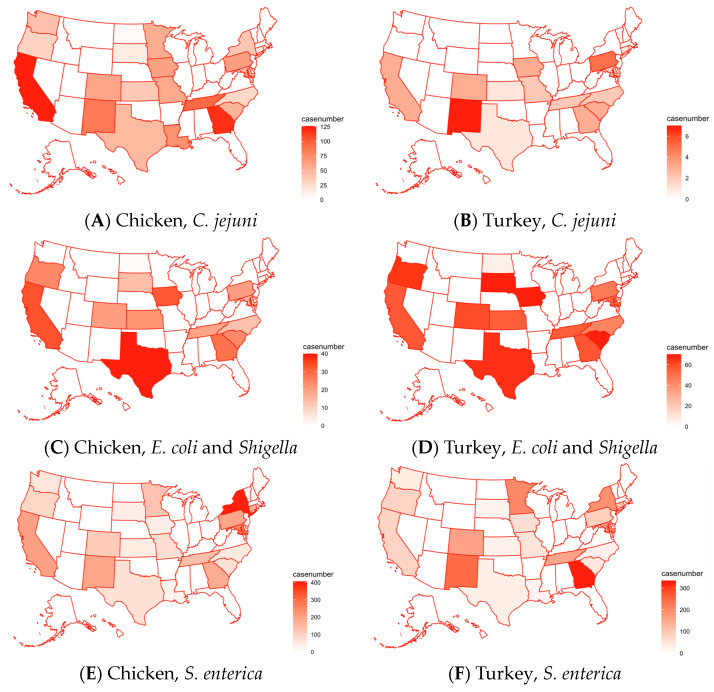
The time profiles of pathogen cases in chicken (the left column) and turkey (the right column) across the U.S., including only the states with data: (**A**) chicken, *C. jejuni*; (**B**) turkey, *C. jejuni*; (**C**) chicken, *E. coli* and *Shigella*; (**D**) turkey, *E. coli* and *Shigella*; (**E**) chicken, *S. enterica*; (**F**) turkey, *S. enterica.* Refer to Appendix A for a map of the U.S. with state names.

As shown in Figure 2, *C. jejuni* was much more common in chicken samples than in turkey samples. Figure 3 shows the distribution of these pathogens in the U.S. The higher-concentration areas included California and Georgia, reaching 125 cases. However, in turkey samples, *C. jejuni* was a lot less common, with the greatest number of cases per state being seven, as found in New Mexico (Figure 3A,B). *E. coli* and *Shigella* were found to be most common in turkey, as shown on the map. The map shows that *E. coli* and *Shigella* in both turkey and chicken (Figure 3C,D) were not evenly distributed, being a lot more concentrated in Texas. As for *S. enterica*, it was the most common pathogen found in chicken and turkey, with the cases for each organism reaching above 200. *S. enterica* was found throughout the U.S. in both chicken and turkey samples, with 100–200 cases in many states (Figure 3E,F). A higher concentration of cases of *S. enterica* in chicken samples can be seen in the New York–Pennsylvania–Connecticut region, likely signaling a spreading. In turkey samples, higher concentrations lay in New Mexico/Colorado, and in Georgia. A similar pattern of spreading of *S. enterica* existed in turkey samples in the same region as chicken, but not to as high a degree.

**Figure 4:**
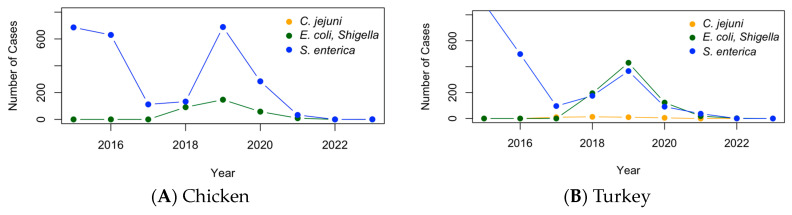
The time profile of pathogen cases in chicken (**A**) and turkey (**B**) in the U.S. from 2015 to 2023, tracking *C. jejuni*, *E. coli*, *Shigella*, and *S. enterica*.

The time profiles demonstrating the change in the levels of pathogens are shown in Figure 4. In 2015, or pre-2015, there was an outbreak of salmonella in both chicken and turkey. It had relaxed by 2017, though there was another outbreak that followed, in 2019. This time, the outbreak was greater in chicken than in turkey. Looking at *C. jejuni*, in turkey, the levels were fairly constant, remaining very low throughout. From around 2017–2019, the *Campylobacter* detection levels were fairly high in chicken compared to the levels shown in turkey. For *E. coli* and *Shigella*, there was also some sort of outbreak in poultry in around 2018–2019. The pathogens were found at higher levels in turkey than in chicken. The levels of all the pathogens calmed down by 2022.

### 3.2. Antimicrobials with Detected Resistance in Pathogens Isolated from U.S. Poultry

**Figure 5:**
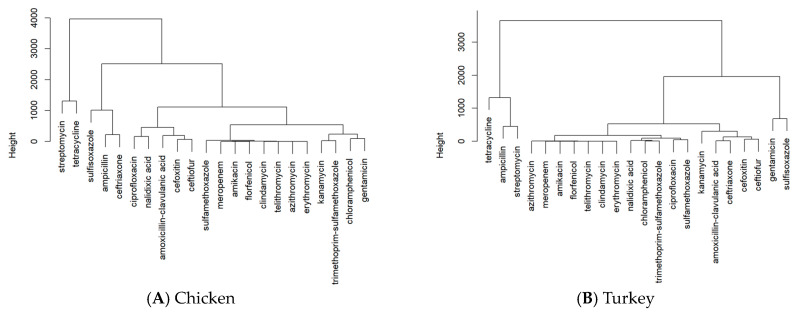
Results of the hierarchical clustering on the antimicrobials with detected resistance by pathogens isolated from U.S. chicken (**A**) and turkey (**B**).

Since there are quite a few antimicrobials in the dataset, some of them are lumped together in the PCA graphs. The hierarchical clustering results are thus mainly shown in Figure 5 for a better illustration. The outlier antimicrobials from the PCA can be identified and confirmed by the clustering results. Based on Figure 5, tetracycline is the antimicrobial that most pathogens in chicken and turkey have resistance against. The next most resisted antimicrobial is streptomycin. For the pathogens found in chicken samples, the third most resisted antimicrobial is sulfisoxazole, followed by ampicillin and nalidixic acid. In turkey samples, the third most resisted antimicrobial is ampicillin, followed by sulfisoxazole and gentamicin.

**Figure 6:**
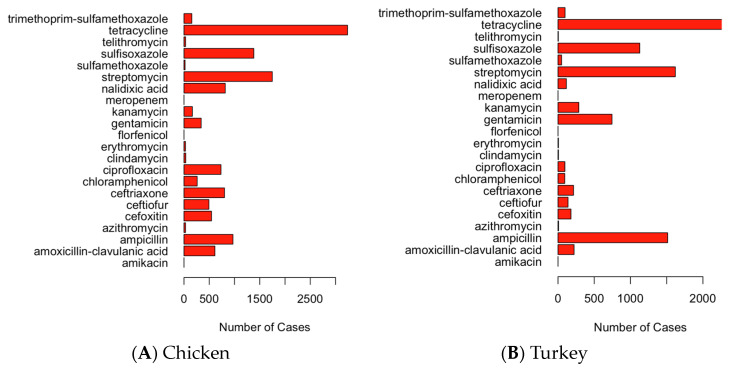
Bar graphs of the number of pathogen samples with resistance to a certain antimicrobial in the U.S.: (**A**) chicken; and (**B**) turkey.

The bar plots shown in Figure 6 indicate that most cases of antimicrobial resistance in pathogen samples from both chicken and turkey are associated with tetracycline, streptomycin, sulfisoxazole, ampicillin, nalidixic acid, and gentamicin. Notably, the third and fourth most common pathogen samples resistant to a specific antimicrobial differ between chicken and turkey: in chicken, sulfisoxazole ranks third and ampicillin fourth, while in turkey, this order is reversed. For the fifth most common antimicrobial resistance, nalidixic acid is prevalent in chicken, while gentamicin is more common in turkey.

**Figure 7:**
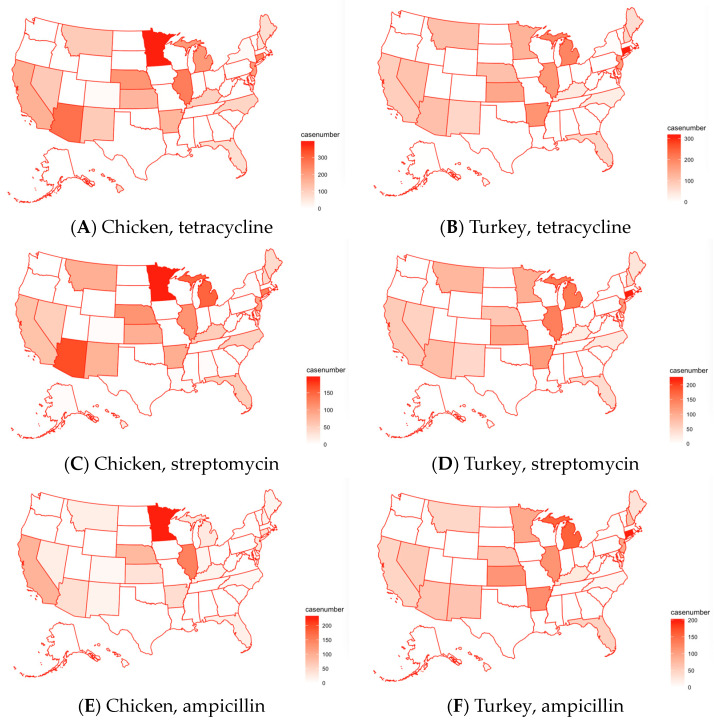

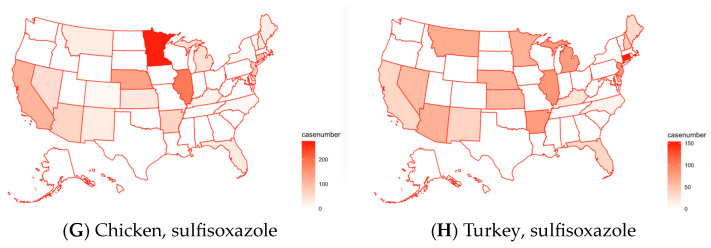
Maps of the distribution of samples with detected resistance to the top four antimicrobials in chicken (the left column) and turkey (the right column) in different states in the U.S.: (**A**) chicken, tetracycline; (**B**) turkey, tetracycline; (**C**) chicken, streptomycin; (**D**) turkey, streptomycin; (**E**) chicken, ampicillin; (**F**) turkey, ampicillin; (**G**) chicken, sulfisoxazole; and (**H**) turkey, sulfisoxazole. Refer to Appendix A for a map of the U.S. with state names.

Due to space constraints, Figure 7 presents the geometric distribution of only the top four antimicrobials exhibiting resistance in pathogens isolated from U.S. poultry. Figure 7A,B illustrate the distribution of tetracycline cases in chicken and turkey across various regions in the U.S. Although both graphs indicate similar regional distributions, there is a notable difference in concentration. Tetracycline cases in chicken are more concentrated in Minnesota, Arizona, and Illinois, whereas cases in turkey are more concentrated in the Midwest and are much more evenly spread out across the country. Figure 7C,D illustrate the distribution of streptomycin-resistant samples in chicken and turkey across various regions in the U.S. While both graphs indicate similar regional distributions, there is a notable difference in concentration (i.e., case numbers). It can be inferred that streptomycin, in comparison to tetracycline, is commonly used in the same areas. In terms of ampicillin, the number of cases of AMR involving ampicillin for chicken is greatly decreased in Arizona compared to the cases of streptomycin and tetracycline (Figure 7E,F). On the other hand, the concentration of the cases of streptomycin, tetracycline, and ampicillin remains constant for the maps of turkey. Ampicillin cases are notably concentrated in Massachusetts but show an even higher concentration in Michigan. There are more cases of ampicillin resistance than chicken-related cases in most areas, with Minnesota being an exception. Sulfisoxazole cases are heavily concentrated in the coastal Mideast for turkey, with significantly fewer cases in the Midwest compared to chicken (Figure 7G,H). In contrast, there is a large concentration of sulfisoxazole cases for chicken in Minnesota.

**Figure 8:**
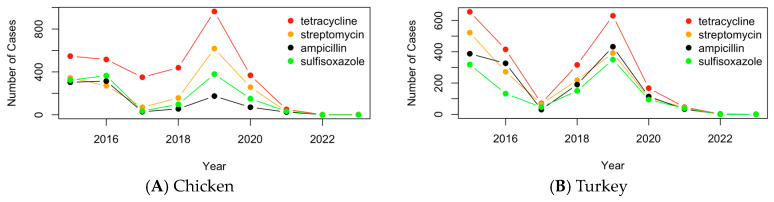
The time profile of the number of the cases of resistance for the top four antimicrobials detected in chicken (**A**) and turkey (**B**).

As seen in Figure 8A, the cases of resistance for the top four antimicrobials detected in chicken drop from 2015 to 2017, before rapidly increasing to their highest points in 2019 and then rapidly decreasing to near zero cases. Figure 8B and the cases of resistance for antimicrobials detected in pathogens isolated from turkey are relatively more straightforward than chicken. Cases rapidly decline from 2015 to 2017 and then rapidly increase in 2019. Cases then rapidly decrease to near zero cases by 2023. In general, both chicken and turkey samples follow a similar trend, with that being the rapid increase in 2019 followed by a rapid decrease to near zero.

### 3.3. Genes Involved in Antimicrobial Resistance of Pathogens Isolated from U.S. Poultry

**Figure 9:**
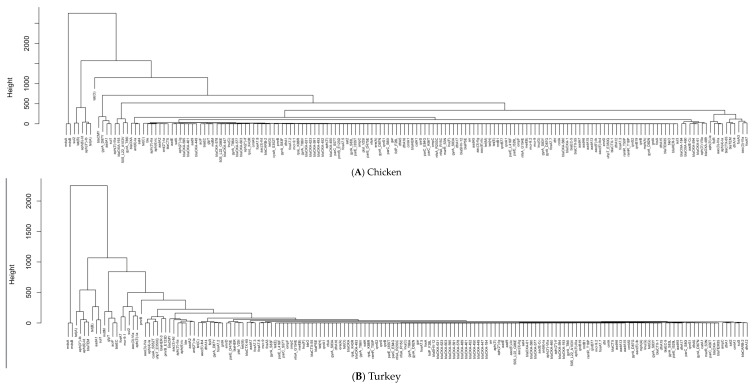
Hierarchical clustering of AMR genes detected in (**A**) chicken and (**B**) turkey.

**Figure 10:**
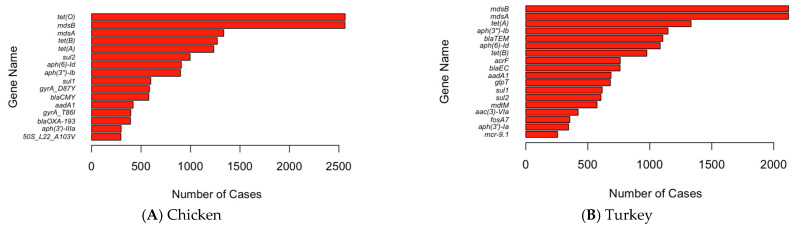
Cases with the individual AMR genes detected in chicken (**A**) and turkey (**B**).

Due to the large number of genes in the dataset, certain genes are lumped into un-distinguishable groups in the two-dimensional PCA plots. Therefore, only the results from the hierarchical clustering are shown in Figure 9 to illustrate the similarity of these genes in terms of their detection frequencies. The genes were compared with the outlier genes shown in the PCA plots (e.g., *tet(O)*, *mdsB*, *mdsA*, *tet(B)*, and *tet(A)*) to confirm their important role in the antimicrobial resistance of pathogens detected in U.S. chicken and turkey. The dendrogram generated from the clustering analysis identified six primary clusters using the elbow method, with genes like *tet(O)*, *mdsB*, *mdsA*, *tet(B)*, and *tet(A)* being identified as important genes. These important genes were confirmed for their high occurrence in the bar plot (i.e., Figure 10), in which the occurrence cases of antimicrobial resistance detected in the dataset were plotted. It seems that the most common genes between cases in chicken and turkey are very similar, with three genes that occur in both of the top five (*tet(A)*, *mdsA*, and *mdsB*). This most likely indicates the importance of genes to develop. Additionally, the differences in the next two most common genes indicate a difference between the pathogens contaminating turkey and chicken and the environments the pathogens experience.

**Figure 11:**
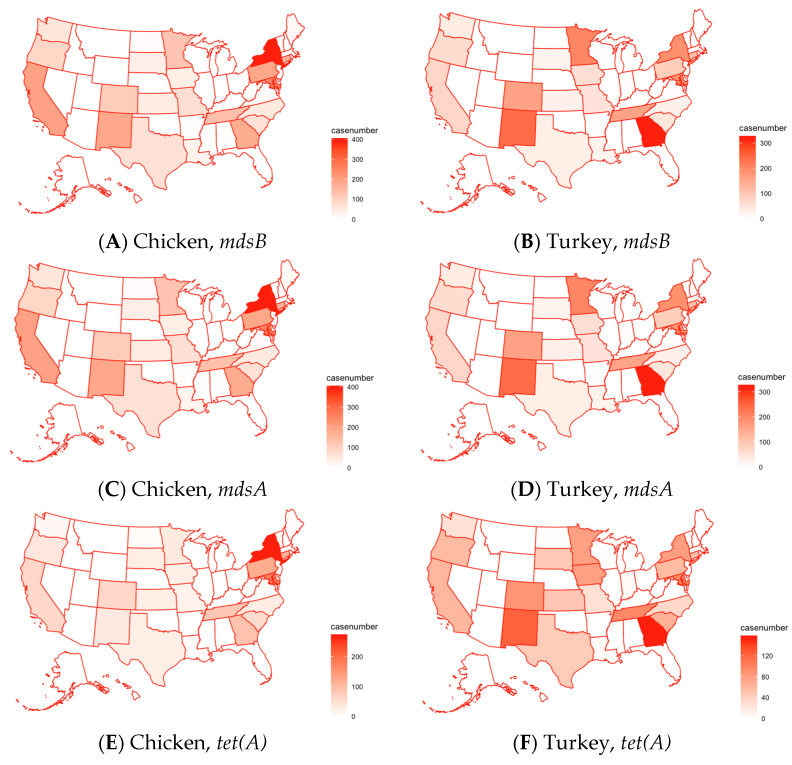

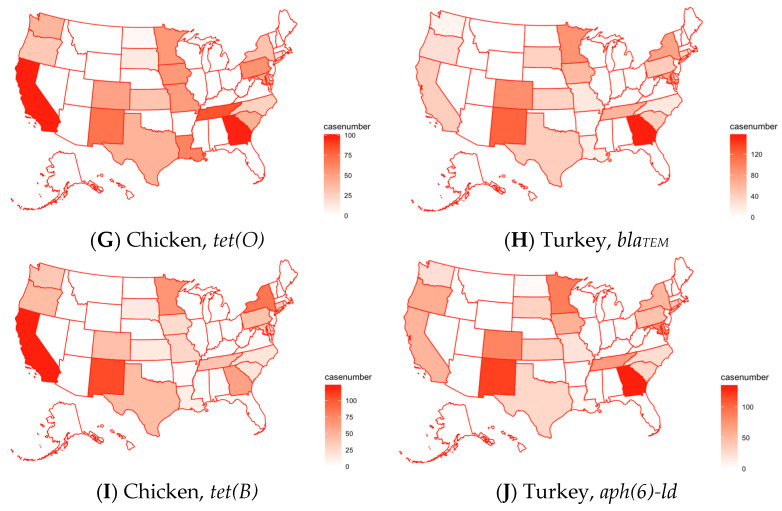
Distribution of the top five antimicrobial resistance genes in different states in the U.S. for pathogens isolated from chicken (the left column) and turkey (the right column): (**A**) Chicken, *mdsB*; (**B**) Turkey, *mdsB*; (**C**) Chicken, *mdsA*; (**D**) Turkey, *mdsA*; (**E**) Chicken, *tet(A)*; (**F**) Turkey, *tet(A)*; (**G**) Chicken, *tet(O)*; (**H**) Turkey, *bla_TEM_*; (**I**) Chicken, *tet(B)*; (**J**) Turkey, *aph(6)-ld*. Refer to Appendix A for a map of the U.S. with state names.

Figure 11A–J below show the geographic distribution of the five most common genes detected in the organisms found in chicken and turkey. For turkey cases, the genes that appear most frequently show up commonly in cases from Georgia and New Mexico, with very little exception. This distribution is consistent across all five genes, which suggests a regional hotspot for antimicrobial resistance genes in turkeys in these states. For genes most common in chicken cases, three are predominantly found in New York, though two others are most prevalent in California. Overall, the genes in chicken cases are more represented all over the United States, while important genes in turkey cases are mostly concentrated in Georgia and New Mexico.

**Figure 12:**
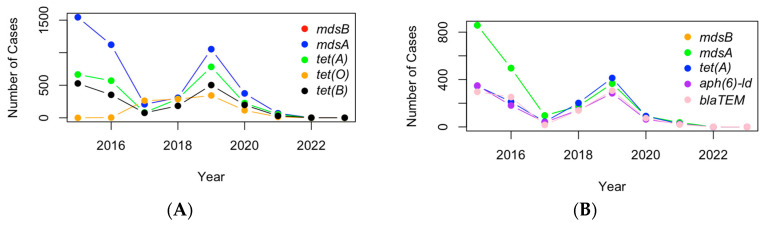
The cases of the top five AMR genes detected in U.S. chicken (**A**) and turkey (**B**).

The time profiles of the most common genes are quite similar between turkey and chicken (Figure 12). They both peak in the year 2019 and dip in 2017. This pattern suggests that there may have been an increase in either the prevalence of these genes or the detection and reporting of AMR cases during 2019. Such a peak could be the result of an outbreak or a change in sampling practices that year. In turkeys, the data are more compact and consistent across the different genes, which indicates that the prevalence of these genes is relatively stable over time. In contrast, the chicken data show much more variability, with fluctuations in the number of cases year to year.

## 4. Discussion

The results of this study provide important insights into antimicrobial resistance patterns in poultry across different regions of the U.S. *S. enterica* was the most commonly pathogen isolated in both chicken and turkey samples, consistent with previous studies identifying *S. enterica* as a dominant contributor to foodborne illnesses and AMR in poultry [34]. Our analysis indicates a significant outbreak of *S. enterica* in 2019, likely centered in the Northeast, with higher concentrations in states such as New York, Connecticut, Pennsylvania, and Maryland. This outbreak was particularly prominent in chicken samples, although similar trends were observed in turkey. Outbreaks in other regions, including California, Georgia, New York, and Minnesota, reflect areas where larger human populations may be at greater risk of pathogen exposure through food sources. The regional prevalence of *C. jejuni* in chicken, especially in California and Georgia, aligns with findings in [35,36], which linked high-density poultry farming to increased *C. jejuni* presence. These regional variations highlight the influence of local agricultural practices on the pathogen distribution and AMR patterns. Although *S. enterica* serotype data could have provided further insights into AMR trends, this information was unavailable in some samples and was excluded from the analysis.

By utilizing PCA graphs, hierarchical clustering, bar graphs, and maps, it is evident that tetracycline is the antimicrobial to which most pathogens in both chicken and turkey samples show resistance, followed by streptomycin. However, there are differences in the ranking of other antimicrobials between the two types of samples. In chicken samples, sulfisoxazole is the third most resisted antimicrobial, followed by ampicillin and nalidixic acid. In turkey samples, ampicillin ranks third in resistance, followed by sulfisoxazole and gentamicin. A comparison of the PCA graphs with the bar graphs reveals that the antimicrobials appearing as outliers tend to be those with the highest resistance levels in both chicken and turkey pathogen samples. This analysis highlights the variability in the antimicrobial resistance patterns between pathogens found in chicken and turkey, particularly in the ranking of the most resisted antimicrobials. The graphs show that antimicrobial resistance cases in chicken are often concentrated in specific regions, such as Minnesota, while in turkey, the cases are more evenly distributed across the Midwest and other areas of the U.S. Overall, while the regional distribution of cases in chicken and turkey is similar, the concentration of cases varies, with chicken showing more localized clusters and turkey having a broader spread.

The most common AMR genes between cases in chicken and turkey were similar, with three genes that occurred in the top five genes twice (*tet(A)*, a tetracycline efflux pump found in many species of Gram-negative bacteria; *mdsB*, a membrane transporter of mdsABC, a part of the Multidrug and Toxic Compound Extrusion (MATE) family of efflux pumps, and *mdsA*, a membrane fusion protein of MdsABC). The mdsABC complex provides a variety of resistances, mostly including beta-lactam antimicrobials. This complex is only found in *S. enterica*. In the chicken population, the gene *Tet(O)*, a ribosomal protection protein that specifically removes tetracycline that inhibits protein production (commonly found in Gram-positive and Gram-negative bacteria) was identified as the most significant contributor to AMR. Previous studies [37,38] have highlighted *tet(O)* as a determinant of tetracycline resistance in agricultural settings. In the turkey population, the *bla_TEM_* and *aph(6)-ld* genes are among the top five AMR genes. The *bla_TEM_* gene produces extended-spectrum beta-lactamases, commonly seen in Gram-negative bacteria. The *aph(6)-ld* genes code for the catalysis and deactivation of aminoglycosides, most notably streptomycin and gentamicin.

The time profiles of the antimicrobial-resistant pathogens, genes, and antimicrobials in U.S. poultry between 2015 and 2023 show notable trends. *Salmonella enterica* was the most prevalent pathogen in both chicken and turkey, peaking in 2015 and 2019. In chicken, *C. jejuni* was more common, showing elevated levels from 2017 to 2019, while *E. coli* and *Shigella* were more prevalent in turkey during 2018–2019. These trends were mirrored in key AMR genes, such as *tet(A)*, *mdsA*, and *mdsB*, which also peaked in 2019 and decreased significantly by 2022. Similarly, resistance to antimicrobials like tetracycline, streptomycin, and sulfisoxazole followed the same pattern, with a spike in 2019 and a decline by 2022. This alignment across pathogens, genes, and antimicrobials suggests a widespread impact of AMR outbreaks and possible changes in the pathogen spread or detection practices during this period. The decline in AMR cases by 2022 may be attributed to effective mitigation efforts, such as improved biosecurity measures, changes in antimicrobial use, or stricter regulations in the poultry industry. However, it is also possible that the COVID-19 pandemic contributed to this decline by reducing detection or measurement efforts due to disruptions in routine surveillance and laboratory capacities. More data are needed to determine the true reason for the declining trend and to clarify the extent to which each factor influenced the observed patterns.

Antimicrobial resistance in poultry may originate primarily from the widespread use of antimicrobials in farming, especially for growth promotion and disease prevention. This overuse creates selective pressure, encouraging the survival of resistant strains like *Salmonella*, *Campylobacter*, and *E. coli*. Over time, these bacteria acquire and share resistance genes, such as *tet(A)* and *mdsB*, which facilitate their ability to withstand antimicrobial treatments. The future implications of AMR are significant, as the rise in resistant strains can make common treatments ineffective and lead to more severe and difficult-to-treat infections in humans. This not only complicates medical care but also has broad public health implications and causes a substantial economic burden on healthcare systems. This study calls for stricter regulations on antimicrobial usage and a coordinated global response through initiatives like One Health to combat the spread of resistance across human, animal, and environmental sectors.

In this study, several limitations arise due to the nature of the dataset used. First, *E. coli* and *Shigella* spp. were grouped together in Figure 2, as this is how the NCBI Pathogen Isolates Browser organizes the data. This grouping likely reflects the close genetic relationship between these species, which makes them difficult to distinguish solely through genomic data. This simplification, while useful for analyzing broad trends in AMR and gastrointestinal diseases, may overlook important distinctions between the two pathogens. Second, the dataset did not differentiate between avian pathogenic *E. coli* (APEC) and commensal *E. coli* in poultry. This lack of detail limits our ability to draw more specific conclusions about the role of different *E. coli* types in poultry antimicrobial resistant patterns. Finally, while the NCBI Pathogen Isolates Browser did provide general source information (e.g., chicken breasts and chicken thighs), detailed metadata on the origin of isolates, such as the collection method (swabs or other sample types), were limited in the data. These factors should be considered when interpreting the results.

## 5. Conclusions

This study highlights the significant public health and economic burden posed by foodborne pathogens and antimicrobial resistance in U.S. poultry, particularly in chicken and turkey. By analyzing over 6900 antimicrobial-resistant pathogen isolates, the findings show that *Salmonella enterica*, *Campylobacter jejuni*, *E. coli*, and *Shigella* are the primary pathogens involved in antimicrobial resistance, with *S. enterica* being the most prevalent. Tetracycline and streptomycin were identified as the most resisted antimicrobials, with AMR genes such as *tet(A)*, *mdsA*, and *mdsB* playing a key role in the resistance patterns. The spatial distribution of these pathogens, AMR genes, and antimicrobials revealed localized clusters of resistance, with the *S. enterica* concentrations highest in regions like New York, Pennsylvania, and Georgia, while resistance to tetracycline was notably concentrated in Minnesota for chicken and more evenly distributed across the Midwest for turkey. Notably, the resistance levels peaked in 2019, followed by a sharp decline by 2022, which may have been driven by a combination of improved biosecurity measures and regulatory changes. In addition, the potential impact of the COVID-19 pandemic on AMR surveillance and detection cannot be overlooked. During the pandemic, disruptions to routine surveillance activities and laboratory capacities likely led to reduced monitoring of AMR cases, as resources were diverted to manage the health crisis. Antimicrobial usage might have decreased in some agricultural settings due to supply chain disruptions, and the pandemic also led to delays in pathogen detection and reporting, which could explain the observed decline in resistance levels during this period. It is crucial to conduct further research to disentangle the effects of reduced surveillance from actual improvements in AMR control during this time. This study underscores the need for comprehensive and continuous AMR monitoring, policy reform, and responsible antimicrobial use to address the growing threat of AMR in the poultry industry and protect public health.

## Data Availability

The data will be provided upon request.

## Appendix A

Map of the U.S. with state names (https://upload.wikimedia.org/wikipedia/commons/a/a5/Map_of_USA_with_state_names.svg), accessed on 19 October 2024.
